# A Mobile App–Based Individualized Nonpharmacological Intervention for Behavioral and Psychological Symptoms in Dementia: Pilot Randomized Controlled Trial

**DOI:** 10.2196/79469

**Published:** 2026-04-07

**Authors:** Eunhee Cho, Minhee Yang, Sinwoo Hwang, Eunkyo Kim, Jungwon Cho, Min Jung Kim

**Affiliations:** 1 Mo-Im Kim Nursing Research Institute, College of Nursing, Yonsei University, 50-1, Yonsei-ro, Seodaemun-gu, Seoul, Republic of Korea, 82 2-2228-3344, 82 2-2227-8303; 2 Army Cadet Military School, Goesan, Republic of Korea; 3 College of Nursing and Brain Korea 21 FOUR Project, Yonsei University, Seoul, Republic of Korea; 4 College of Nursing, Ewha Womans University, Seoul, Republic of Korea

**Keywords:** behavioral and psychological symptoms of dementia, dementia, mobile app, nonpharmacological intervention, randomized controlled trial

## Abstract

**Background:**

Behavioral and psychological symptoms of dementia (BPSD) are common and negatively impact both individuals with dementia and their families. Although nonpharmacological interventions are recommended as the first-line treatments, their use in community settings is limited by access and caregiver resources. Existing approaches are often not individualized and rely on institutional or clinician-led delivery.

**Objective:**

We developed a caregiver-initiated and individualized multimodal mobile app. The app delivers tailored nonpharmacological interventions—such as music therapy, exercise, and reminiscence therapy—based on each user’s preferences and functional abilities. This study aimed to evaluate the effectiveness of this mobile app–based intervention in reducing BPSD in community-dwelling persons living with dementia.

**Methods:**

This study used a single-blinded randomized controlled trial design. Participants were recruited from an outpatient clinic of a tertiary hospital, a dementia care center, and 5 home care service centers. A total of 36 dyads participated, each comprising a community-dwelling person living with dementia aged 60 years or older and their primary family caregiver. The dyads were randomly allocated to either the intervention or control group. The intervention group received a caregiver-initiated, multimodal, mobile app–based individualized intervention for 4 weeks, whereas the control group continued with usual care. The primary outcomes were overall BPSD, agitated behavior, and depression. The secondary outcomes were nighttime sleep efficiency and caregiver competency in managing BPSD. Assessments were conducted at baseline, immediately after the intervention, and at a 2-week follow-up.

**Results:**

Of the 36 randomized dyads, 33 were included in the final analysis. Although the intervention group showed greater reductions in overall BPSD, agitated behavior, and depression after the intervention, no significant group-by-time interaction effects were observed in the total sample. In the subgroup analysis of participants with clinically significant baseline BPSD, a statistically significant improvement in overall BPSD was found in favor of the intervention group (*β*=–12.885, 95% CI –24.530 to –1.240; *P*=.03). No significant effects were observed for either nighttime sleep efficiency or competence in the management of BPSD.

**Conclusions:**

A mobile app–based individualized intervention may offer a flexible, caregiver-initiated approach to managing BPSD in home-care settings. While overall effects were limited, exploratory subgroup findings provided meaningful insights, indicating potential benefits for those with higher baseline symptom severity. The results highlight the need for further research on adaptive personalization and optimized intervention delivery to enhance the clinical effectiveness of digital dementia care.

## Introduction

Cognitive decline is a common symptom of dementia, with behavioral and psychological symptoms of dementia (BPSD) recognized as the core features of the condition [1]. The nature of BPSD varies depending on the type and stage of the condition; however, these symptoms can appear at any time [1]. According to recent literature, nearly 90% of persons living with dementia experience BPSD [2]. Among these, symptoms such as apathy, depression, anxiety, irritability, agitation, and aggression are commonly observed in persons living with dementia [3], and often coexist with sleep disturbances, which are highly prevalent in this population [4-6]. In particular, a person living with dementia frequently exhibits reduced nighttime sleep efficiency and disrupted rest–activity rhythms, characterized by fragmented nocturnal sleep and frequent nighttime awakenings [4,5]. These disturbances are not simply co-occurring conditions but interact bidirectionally with BPSD, jointly contributing to increased mortality [7] and decreased quality of life [8]. Furthermore, BPSD and nocturnal sleep disruptions significantly increase caregiver burden [8-11] and care costs [8]. Therefore, implementing effective interventions targeting BPSD has become critical in dementia care. Such interventions can alleviate caregiver burden at the individual level and mitigate the broader societal impact of dementia care.

Nonpharmacological interventions are recommended as a first-line strategy for managing BPSD owing to their comparable efficacy to pharmacological treatments while minimizing adverse effects [12,13]. A wide range of nonpharmacological approaches has been developed to address the cognitive, psychological, social, and environmental dimensions of dementia care [12-14]. Interventions such as music therapy, exercise, and reminiscence therapy have demonstrated effectiveness in managing BPSD [14-17] and may be applied to support their prevention and management. Despite the growing emphasis on nonpharmacological interventions for BPSD, the COVID-19 pandemic has exacerbated the exclusion of community-dwelling persons living with dementia from essential care services. In particular, access to nonpharmacological activities was restricted by global social distancing measures [18-20]. These challenges persist beyond the pandemic, particularly among family caregivers, who often face limited access to therapy, logistical challenges in scheduling sessions, insufficient funding, and poor local accessibility [21].

In light of the constraints on home-based nonpharmacological interventions during and after the pandemic, digital approaches for remote delivery have gained growing importance [22]. Digital technologies, such as virtual reality, robotics, and mobile apps, have been widely applied to deliver diverse nonpharmacological interventions for persons living with dementia. These technologies have enabled activity-based programs, exercise, interactive activities, and coaching or counseling support [23-25]. A systematic review found that reminiscence therapy delivered through mobile apps enhanced social interaction [23], and a pet robot in long-term care settings helped alleviate BPSD [24]. However, most of these digital tools have been implemented in long-term care settings or require operation by clinicians or trained staff [25]. Among these, artificial intelligence–based cognitive stimulation programs and digital reminiscence therapies have been delivered by care staff in institutional environments [26,27]. A range of apps and digital platforms enables clinicians and formal caregivers to assess, record, and manage BPSD, with limited applicability to home settings [28-30]. Although mobile apps are also used to provide family caregivers with training on disease-related information, caregiving skills, and coping strategies [31,32], adherence to nonpharmacological interventions remains low [21,33]. Moreover, existing apps rarely enable family caregivers to directly deliver these interventions in real-world home settings, highlighting a critical gap in current digital care approaches. While these interventions have shown beneficial effects on BPSD, their impact could be further enhanced through greater individualization [25,26,34]. Therefore, there is a need for enhanced digital tools to support caregiver-led interventions and tailor strategies to the diverse contexts of persons living with dementia.

To address this gap, we developed a mobile app to empower family caregivers to deliver tailored interventions in real-world home settings. The app’s usability and user acceptability were evaluated during its developmental phase among persons living with dementia and their caregivers [35]. The app guides caregivers through a structured process that includes the assessment of symptoms and potential triggers and recommends tailored nonpharmacological strategies that caregivers can implement. Because BPSD management often exceeds the competence of family caregivers, the app was also designed to gradually build their skills and confidence in delivering these interventions. Building on these findings, we designed a randomized controlled trial to assess the effectiveness of this mobile app–based intervention for persons living with dementia in a community setting. We hypothesized that it would alleviate the severity and frequency of BPSD compared with usual care. The objective of this randomized controlled trial was to evaluate the effectiveness of a mobile app that delivers individualized nonpharmacological interventions for reducing BPSD. The study also examined its effects on nighttime sleep efficiency and caregiver competence.

## Methods

### Study Design and Setting

**Figure 1.**
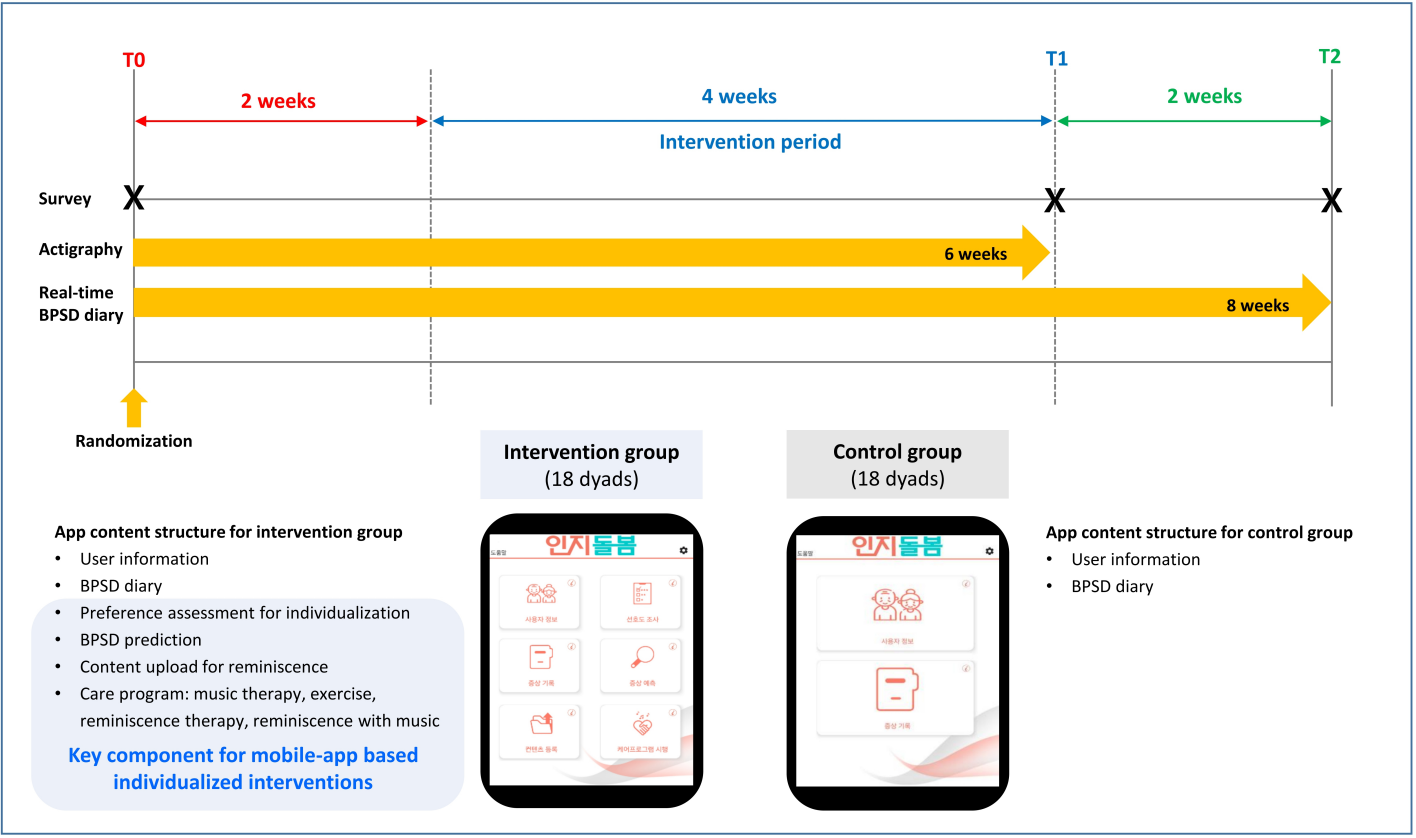
Study timeline and app components by group. BPSD: behavioral and psychological symptoms of dementia.

This was a single-blind, parallel, randomized controlled trial designed to compare mobile app–based individualized nonpharmacological interventions with usual care. Participants were recruited from the neurology outpatient clinic of a tertiary hospital, a regional dementia care center, and 5 home care service centers. Figure 1 presents an overview of the study timeline and app components for each group.

Trained research assistants visited participants to collect outcome data and baseline information according to the study timeline and provided education on app use. They remained available to answer any questions and ensure completeness while family caregivers completed self-administered paper questionnaires. Outcome assessments were conducted at baseline (T0), 4 weeks postintervention (T1), and at 2 weeks of follow-up (T2). The trial was prospectively registered with the Clinical Research Information Service (registration number: KCT0008713). The study protocol has been published previously [36].

### Participants

The study sample included persons living with dementia in a community setting and their family caregivers as a dyad. Eligibility was assessed by trained research assistants. The inclusion criteria for persons living with dementia were (1) aged 60 years or older, (2) any type of dementia, (3) Korean version of the Mini-Mental State Examination score <24, (4) exhibited BPSD at least once per week, and (5) lived in Seoul or Gyeonggi-do Province with their family caregivers. The inclusion criteria for family caregivers were (1) primary caregivers of persons living with dementia, and (2) no difficulties in reading and writing Korean. The participants were enrolled between October 2023 and March 2024, based on the eligibility criteria.

### Ethical Considerations

This study was approved by the institutional review board of Yonsei University Health System (IRB number 4-2023-0660). Written informed consent was obtained from both persons living with dementia and their primary family caregivers after a detailed explanation of the study objectives and procedures. In cases where persons living with dementia were unable to provide consent due to impaired decision-making capacity, consent was obtained from a surrogate decision-maker. Given the dependency and vulnerability of the study population, special ethical considerations were applied throughout the study period. To ensure confidentiality, all participants were assigned identification numbers, and access to the collected data was restricted to the research team only. A small token of appreciation was provided based on the study participation period. Participants who completed the entire study received approximately US $146 (KRW ₩ 200,000) per dyad.

### Sample Size

The sample size was calculated using Cohen’s power analysis (repeated measure analysis of variance within factor: *α*=.05, power level=0.8, effect size=0.25, groups=2, and number of measurements=3). A minimum sample size of 28 participants was required to achieve a statistical power of 0.80. As the intervention was primarily implemented by caregivers, an anticipated 30% attrition rate was applied, based on previous intervention studies with dementia caregivers [37,38]. Accordingly, 36 patient–caregiver dyads were recruited from both groups.

### Randomization and Blinding

Participants were randomly assigned to the intervention and control groups in a 1:1 allocation ratio. A researcher who was not in contact with the participants created a randomization list using a computer program (STATA version 13.1; StatCorp LP). Block randomization (with a block size of 4 to assign patients to 9 groups) was used to evenly allocate the dyads to the intervention and control groups. The generated randomization list was sealed in an envelope and stored. After participants provided consent and the study commenced, group allocation was disclosed only to the researcher responsible for data collection and mobile app training. Persons living with dementia and their caregivers were not informed of their group allocation to minimize bias. Both the intervention and control groups used the same mobile app interface and wore actigraphy devices during the study period. The only distinction was that the intervention group used both symptom diaries and individualized nonpharmacological interventions through the app, whereas the control group used only symptom diaries via the app.

### Intervention

All participants were provided with a tablet device (Galaxy Tab A8; Samsung) preinstalled with a mobile app. Both the intervention and control groups were instructed to record a real-time diary of BPSD throughout the study period using the app, allowing continuous monitoring of symptoms and possible triggers.

Only the intervention group received a 4-week individualized nonpharmacological intervention program via the app. Caregivers were instructed to initiate the intervention at least once daily but could use it more frequently or flexibly according to their needs. The program included 4 types of activities: music therapy, exercise, reminiscence therapy, and reminiscence therapy with music. To enhance individualization, the trained research assistants collected information on personal backgrounds, preferences, and physical functioning at baseline. The app also integrates individualized content, such as family-uploaded photographs, videos, individualized music playlists, and tailored exercise programs. Additionally, the intervention content was updated based on caregivers’ requests to reflect their changing preferences and needs. Family caregivers in the intervention group autonomously selected and initiated interventions based on the app’s recommendations and observed the response of persons living with dementia during the 4-week period.

In addition, the app incorporated a machine-learning–based BPSD prediction model developed and validated in our previous studies [35,39]. The model was trained using data collected from 187 community-dwelling persons living with dementia, with data from 35 others used for external validation. Information collected included demographic data, health status, and personality traits prior to dementia onset. Actigraphy devices tracked participants’ sleep and activity, and caregivers documented daily BPSD symptoms and possible triggers in a symptom diary. Multiple algorithms were tested, including logistic regression, random forest, gradient boosting machine, and support vector machine. Among these, the gradient boosting machine demonstrated the best predictive performance, with average AUC values above 0.8 for most symptoms [39]. Based on these findings, the gradient boosting model was implemented within the mobile app to provide BPSD prediction. Furthermore, we conducted an umbrella review to identify evidence-based nonpharmacological approaches with demonstrated effectiveness for each BPSD to guide the selection of appropriate interventions [14]. Based on this review, we developed evidence-based recommendations, which were integrated into the app. Thus, this app recommends tailored nonpharmacological interventions based on BPSD subtypes predicted through machine learning. Meanwhile, the control group did not receive any nonpharmacological interventions through the app and continued routine care throughout the same period.

### Measurements

Primary outcomes were assessed at 3 time points based on the observation of primary family caregivers. Secondary outcomes regarding nighttime sleep efficiency and caregiver competence in managing BPSD were also collected at baseline and at the 4-week postintervention period.

#### Primary Outcomes

The Korean version of the Neuropsychiatric Inventory (NPI) was used to assess overall dementia-related symptoms. The total score is calculated by multiplying the frequencies and severities of the domains, with a higher score indicating more BPSD [40]. Agitated behaviors were measured using the Korean version of the Cohen-Mansfield Agitation Inventory, which consists of 29 items that assess the frequency of agitated behaviors. The total score ranges from 29 to 203, with higher scores indicating a higher frequency of agitated behavior [41]. Depressive symptoms were evaluated using the Korean version of the Cornell Scale for Depression, which contains 19 items on depressive signs and symptoms [42]. The total score on this scale ranges from 0 to 38, with a higher score indicating a more depressed status.

#### Secondary Outcomes

The activity monitor wGT3X-BT (ActiGraph, LLC), a validated actigraphy device for assessing circadian rhythms in persons living with dementia [43], was used in this study. Persons living with dementia were instructed to wear the wrist-worn actigraphy device as consistently as possible, particularly at night. Sleep data were collected over 2 distinct periods: during the 2 weeks prior to the intervention (baseline period) and throughout the 4-week intervention period. The initial 2-week period was used to collect the data necessary for predicting BPSD patterns and establishing baseline information. The subsequent 4-week period was used to evaluate the effects of the intervention. Nighttime sleep data from these periods were extracted and analyzed to monitor changes in sleep patterns. Sleep efficiency was operationalized as nighttime sleep efficiency, given that it serves as a core indicator of overall sleep quality in persons living with dementia [44]. Previous studies have demonstrated that insufficient nighttime sleep is closely associated with the exacerbation of BPSD [4-6,45] and increased caregiver burden [11], highlighting its clinical relevance in dementia care. Nighttime sleep efficiency was calculated for the period between 8 PM and 8 AM as the ratio of total sleep time to time in bed, consistent with the operational definitions used in previous studies [5].

Our app was designed to provide a comprehensive care framework that includes symptom assessment, prediction of BPSD, recommendation of tailored nonpharmacological strategies, and delivery of interventions. This structured process was expected to enhance caregivers’ competence in managing BPSD, thereby contributing to the effectiveness and sustainability of the intervention. Accordingly, caregiver competence was assessed using the competence scale in managing BPSD [46]. This is a self-report tool for measuring knowledge, skills, and attitudes in the management of BPSD. In addition to the 28 core items, an additional question assessed family caregivers’ general competency.

### Engagement Measures

As both groups completed BPSD diaries for 8 weeks, the number of diary entries was obtained from in-app logs. During the 4-week intervention period, app usage data in the intervention group were extracted from the logs, including usage frequency and mean usage time for each program. The app dose for each participant was operationalized as the number of days used, the total number of sessions, and the mean session duration. Engagement level was defined as the degree to which the persons living with dementia actively participated in the app-guided nonpharmacological intervention. It was rated by family caregivers after each session on a 5-point Likert scale ranging from very bad (1) to very good (5).

### Basic Information

Sociodemographic variables for persons living with dementia included sex, age, years of education, marital status, and dementia diagnosis. Cognitive function was assessed with the Korean version of Mini-Mental State Examination-2, with scores below 24 out of 30 indicating cognitive impairment [47]. Activities of daily living were measured using the Korean Activities of Daily Living scale [48], a 7-item measure of basic physical functions (eg, dressing, face washing, bladder/bowel control, eating, toilet use, and mobility). Higher scores reflect greater dependence, with scores ≥8 indicating dependency. Sociodemographic variables for family caregivers included sex, age, years of education, and relationship with persons living with dementia. Family caregiver–perceived intimacy was assessed using a single Likert item (“How close would you describe your relationship with the person living with dementia?”) rated on a 5-point scale from very high (1) to very low (5). For analysis, scores of 1‐2 were categorized as high intimacy, 3 as moderate intimacy, and 4‐5 as low intimacy. Caregiver subjective health was assessed with a single 5-point item from very healthy (1) to very unhealthy (5), and for analysis, scores of 1 or 2 were categorized as healthy, and 3 to 5 as moderate-to-unhealthy.

### Statistical Analyses

All statistical analyses were performed using SPSS Statistics for Windows (version 27.0; IBM Corp., Armonk, NY). All statistical tests were 2-tailed with a significance level of 0.05. Descriptive statistics were used to summarize participants’ general characteristics. Baseline homogeneity between the intervention and control groups was assessed using the Pearson chi-square test, Fisher exact test, and the independent *t* test. We applied a mixed-effects model to examine whether changes in outcomes over time differed between the intervention and control groups across the 3 measurement time points (T0, baseline; T1, postintervention; and T2, follow-up). The model included fixed effects for group, time, and their interaction (group×time). The baseline value of each outcome variable was included as a covariate to adjust for initial differences between participants. A subject-level random intercept was specified to account for within-participant correlations arising from repeated measures. Time was modeled as a categorical factor to avoid assuming linearity. In this specification, each group×time coefficient reflects the time-specific difference-in-difference, that is, the between-group difference in change from baseline. This approach enabled us to (1) incorporate all available data under the missing-at-random assumption, (2) model within-subject covariance structures to improve estimation efficiency, and (3) perform an omnibus Wald/F test of overall trajectory differences. This reduced the risk of multiplicity compared with conducting separate per-visit analyses. Furthermore, as BPSD are expected to change gradually over time, alternative covariance structures were compared. Specifically, compound symmetry and first-order autoregressive structures were evaluated, and model fit was compared using the Akaike Information Criterion and the Bayesian Information Criterion. The first-order autoregressive structure provided the better fit and was therefore adopted. Although unstructured and independent structures converged, the final Hessian was not positive definite, and therefore, they were not considered further. To improve the accuracy of inference with a relatively small sample, degrees of freedom were adjusted using the Kenward–Roger method.

In addition, due to heterogeneity in baseline BPSD, we conducted an exploratory subgroup analysis of participants with baseline NPI scores ≥11, based on a commonly used threshold for clinically significant BPSD [49]. This analysis aimed to explore whether the intervention exerted greater effects in participants with more severe baseline symptoms. Within this subgroup, the same linear mixed-effects model including group, time, and group×time was fitted to examine whether the intervention effect differed among participants with higher baseline symptom burden. This model was chosen to ensure consistency with the main analysis and to account for repeated measures while estimating time-specific differences in intervention effects within this clinically meaningful subgroup.

## Results

### Participant Baseline Characteristics

**Figure 2.**
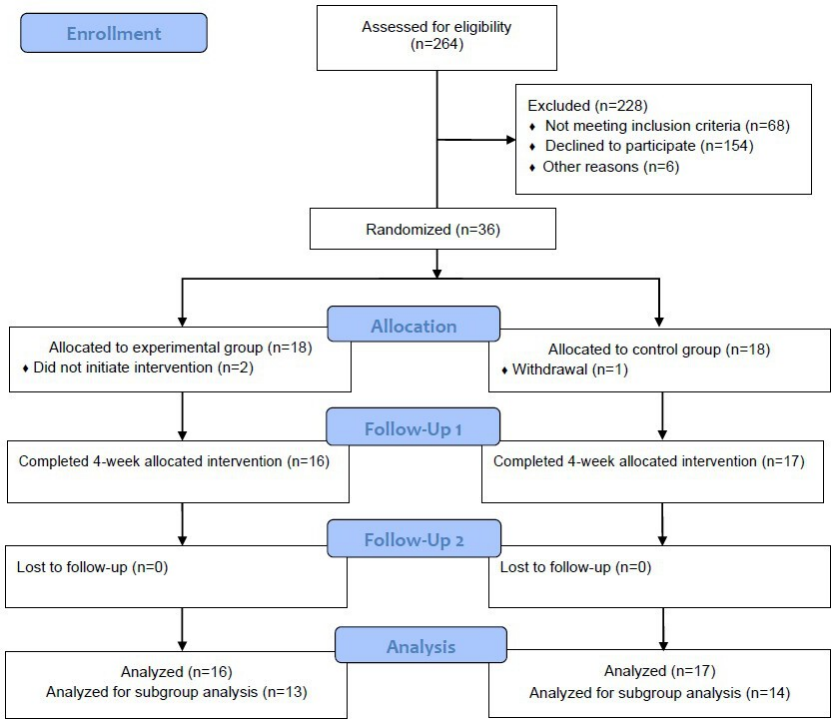
Study flow chart of participants.

Participants were recruited between October 2023 and March 2024, with follow-up being completed by April 2024. As shown in Figure 2, 264 participants were assessed for eligibility across 7 institutions (a neurology outpatient clinic in a tertiary hospital, a dementia care center, and 5 home care service centers). Of these, 36 dyads were enrolled and randomly allocated to either the intervention (n=18) or the control group (n=18). One dyad from the control group withdrew consent for personal reasons before the intervention. Two dyads in the intervention group withdrew because the intervention could not be initiated, as the persons living with dementia refused to participate due to worsening BPSD. Therefore, data from 33 dyads were included in the analysis. The participants’ baseline characteristics are presented in Table 1. The mean age of the participants was 82.70 (SD 7.10) years, and most of them (24/33, 72.7%) were female. A homogeneity test of the persons living with dementia showed no significant differences between the groups at baseline. As shown in Table 2, the mean age of the family caregivers was 65.03 (SD 11.28) years, and most of them (27/33, 81.8%) were female. Family caregivers had provided care for their relatives with dementia for an average of 4.39 (SD 4.37) years, offering approximately 16.32 (SD 8.58) hours of care per day.

**Table 1.** Participants’ characteristics at baseline (N=33).

Characteristics	Total (N=33)	Intervention (n=16)	Control (n=17)	*t* test (*df*)	Chi-square (*df*)	*P* value
Sex, n (%)				—^a^	0.248 (1)	.71^b^
Male	9 (27.3)	5 (31.3)	4 (23.5)			
Female	24 (72.7)	11 (68.8)	13 (76.5)			
Age (years), mean (SD)	82.70 (7.10)	84.19 (6.15)	81.29 (7.81)	−1.178 (31)	—	.25
Marital status, n (%)					0.047 (1)	.83
Married	20 (60.6)	10 (62.5)	10 (58.8)			
Widowed	13 (39.4)	6 (37.5)	7 (41.2)			
Education years, n (%)				—	0.029 (1)	.87
≤6 years	17 (51.5)	8 (50)	9 (52.9)			
>6 years	16 (48.5)	8 (50)	8 (47.1)			
Diagnosis, n (%)				—	0.732 (1)	.48^b^
AD^c^	12 (36.4)	7 (43.8)	5 (29.4)			
Others	21 (63.6)	9 (56.3)	12 (70.6)			
MMSE^d^, mean (SD)	16.67 (5.78)	15.06 (7.12)	18.18 (3.76)	1.556 (22.461)	—	.13
ADL^e^, mean (SD)	12.24 (4.24)	12.38 (4.67)	12.12 (3.94)	−0.171 (31)	—	.87
Overall BPSD^f^, mean (SD)	30.64 (22.48)	30.69 (21.05)	30.59 (24.39)	−0.012 (31)	—	.99
Agitated behavior, mean (SD)	48.24 (18.07)	46.19 (16.53)	50.18 (19.71)	0.628 (31)	—	.54
Depression, mean (SD)	9.88 (6.10)	9.25 (4.70)	10.47 (7.28)	0.576 (27.537)	—	.57
Nighttime sleep efficiency (%), mean (SD)	94.87(2.38)	95.38 (2.73)	94.38 (1.95)	−1.209 (31)	—	.24

a Not applicable.

b Fisher exact test.

c AD: Alzheimer disease.

d MMSE: Mini-Mental State Examination.

e ADL: activities of daily living.

f BPSD: behavioral and psychological symptoms of dementia.

**Table 2.** Family caregivers’ characteristics at baseline (N=33).

Characteristics	Total (n=33)	Intervention (n=16)	Control (n=17)	*t* test (df)	Chi-square (*df*)	*P* value
Sex, n (%)				—^a^	0.007 (1)	>.99^b^
Male	6 (18.2)	3 (18.8)	3 (17.6)			
Female	27 (81.8)	13 (81.3)	14 (82.4)			
Age (years), mean (SD)	65.03 (11.28)	64.88 (11.09)	65.18 (11.80)	0.076 (31)	—	.94
Education years, n (%)				—	0.004 (1)	>.99^b^
≤6 years	4 (12.1)	2 (12.5)	2 (11.8)			
>6 years	29 (87.9)	14 (87.5)	15 (88.2)			
Relationship, n (%)				—	0.047 (1)	.83
Spouse	13 (39.4)	6 (37.5)	7 (41.2)			
Child	20 (60.6)	10 (62.5)	10 (58.8)			
Caregiver–perceived intimacy with persons living with dementia , n (%)				—	0.170 (1)	>.99^b^
High	28 (84.8)	14 (87.5)	14 (82.4)			
Moderate	5 (15.2)	2 (12.5)	3 (17.6)			
Caregiving period (years), mean (SD)	4.39 (4.37)	4.84 (3.48)	3.97 (5.14)	−0.568 (31)	—	.57
Caregiving hours per day, mean (SD)	16.32 (8.58)	15.54 (9.35)	17.05 (8.01)	0.498 (31)	—	.62
Subjective health status, n (%)				—	0.750 (1)	.39
Healthy	16 (48.5)	9 (56.3)	7 (41.2)			
Moderate-unhealthy	17 (51.5)	7 (43.8)	10 (58.8)			
Competence in managing BPSD^c^^d^, mean (SD)	102.48 (13.95)	102.00 (15.41)	103.00 (12.74)	0.196 (29)	—	.85

a Not applicable.

b Fisher exact test.

c Competence in managing BPSD was assessed in 15 control group participants owing to missing data.

d BPSD: behavioral and psychological symptoms of dementia.

### Effects of Mobile App–Based Individualized Interventions on Outcome Variables

Table 3 presents the changes in mean scores and group×time effects for each outcome variable, with baseline scores included as covariates in the linear mixed model analysis. Descriptive trends indicated reductions in overall BPSD, agitated behavior, and depression in both groups after the 4-week intervention. The linear mixed model analysis revealed no statistically significant group×time effects for overall BPSD (*β*=–8.647, 95% CI –18.737 to 1.442; *P*=.09), agitated behavior (*β*=–1.290, 95% CI –7.236 to 4.655; *P*=.67), or depression (*β*=.121, 95% CI –2.794 to 3.037; *P*=.93).

**Table 3.** Effects of the mobile app–based individualized nonpharmacological intervention on outcome variables.

Outcome variables		Intervention (n=16)	Control (n=17)	Estimate^a^ (95% CI)	*P* value
Overall BPSD^b^, mean (SD)					
T1^c^-T0^d^		−11.00 (18.46)	−2.35 (13.13)	−8.647 (−18.737 to 1.442)	.09
T2^e^-T0		−8.44 (20.37)	−3.76 (17.89)	−4.673 (−16.689 to 7.343)	.44
Agitated behavior, mean (SD)					
T1-T0		−1.94 (7.45)	−0.65 (7.56)	−1.290 (−7.236 to 4.655)	.67
T2-T0		−4.38 (10.26)	−3.24 (10.69)	−1.140 (−7.913 to 5.634)	.74
Depression, mean (SD)					
T1-T0		−0.94 (5.69)	−1.06 (4.41)	0.121 (−2.794 to 3.037)	.93
T2-T0		−2.13 (4.88)	−0.59 (3.26)	−1.537 (−4.832 to 1.758)	.36
Nighttime sleep efficiency (%), mean (SD)					
T1-T0		−0.57 (1.42)	−0.52 (1.95)	−0.054 (−1.271 to 1.163)	.93
Competence in managing BPSD, mean (SD)					
T1-T0		6.00 (12.78)	0.07 (12.19)	5.933 (−6.979 to 18.846)	.36

a All models were adjusted for baseline scores as covariates. The control group at baseline (T0) was used as the reference.

b BPSD: behavioral and psychological symptoms of dementia.

c T1: after 4 weeks.

d .T0: baseline

e T2: 2-week follow-up.

At the 2-week postintervention period, overall BPSD increased in the intervention group, while the control group maintained a downward trend. This difference, however, did not reach statistical significance (*β*=–4.673, 95% CI –16.689 to 7.343; *P*=.44). Agitated behaviors continued to decrease in both groups, with no statistically significant interaction effect (*β*=–1.140, 95% CI –7.913 to 5.634; *P*=.74). Depressive symptoms increased in the control group but showed a downward trend in the intervention group. However, no statistically significant group×time effect was observed (*β*=–1.537, 95% CI –4.832 to 1.758; *P*=.36).

Regarding secondary outcomes, no statistically significant group×time interaction effect on nighttime sleep efficiency was observed after the intervention (*β*=−.054, 95% CI −1.271 to 1.163; *P*=.93). Family caregivers’ level of competence in managing BPSD increased in the intervention group; however, the group×time effect was not statistically significant (*β*=5.933, 95% CI –6.979 to 18.846; *P*=.36). No harm was recorded during the study period in either group.

### Subgroup Analysis of Outcomes

Table 4 presents a subgroup analysis among participants with baseline NPI scores ≥11, a cutoff previously identified as indicating clinically significant BPSD. Greater reductions in overall BPSD, agitated behavior, and depression were observed in the intervention group over the study period. However, the linear mixed model analysis controlling for baseline scores showed that only the group×time interaction for overall BPSD was statistically significant after the 4-week intervention (*β*=–12.885, 95% CI –24.530 to –1.240; *P*=.03).

**Table 4.** Subgroup analysis of outcomes in participants with Neuropsychiatric Inventory≥11 at baseline.

Outcome variables		Intervention (n=13)	Control (n=14)	Estimate (95% CI)	*P* value
Overall BPSD^a^, mean (SD)					
T1-T0^b^^c^		−15.38 (16.70)	−2.50 (14.53)	−12.885 (−24.530 to −1.240)	.03^d^
T2-T0^e^		−12.23 (20.30)	−3.86 (19.85)	−8.374 (−22.221 to 5.474)	.23
Agitated behavior, mean (SD)					
T1-T0		−2.08 (8.26)	−0.07 (8.17)	−2.005 (−9.116 to 5.105)	.57
T2-T0		−6.00 (10.60)	−3.14 (11.66)	−2.857 (−10.944 to 5.230)	.48
Depression, mean (SD)					
T1-T0		−1.92 (5.41)	−1.07 (4.68)	−0.852 (−4.073 to 2.369)	.60
T2-T0		−3.31 (3.95)	−0.07 (3.22)	−3.236 (−6.753 to 0.281)	.07
Nighttime sleep efficiency (%), mean (SD)					
T1-T0		−0.57 (1.56)	−0.42 (1.95)	−0.152 (−1.558 to 1.255)	.83
Competence in managing BPSD, mean (SD)					
T1-T0		6.85 (23.76)	0.77 (12.99)	6.077 (−9.423 to 21.576)	.43

a BPSD: behavioral and psychological symptoms of dementia.

b T1: after 4 weeks.

c T0: baseline.

d Statistically significant values (*P*<.05).

e T2: 2-week follow-up.

### Use of Behavioral and Psychological Symptoms of Dementia Diary and App-Based Individualized Nonpharmacological Interventions

During the 4-week intervention period, the frequency and intensity of use varied across participants. The total number of days the intervention was used ranged from 6 to 25, and the total number of sessions ranged from 9 to 45. The average session duration ranged from 11 to 54 minutes. On average, participants engaged with the intervention on approximately 15 days, completing about 22 sessions, with a mean session duration of around 30 minutes.

Tables5  6 present the logs of the BPSD diary records and mobile app–based individualized nonpharmacological interventions. Over the 8-week study period, the total number of diary records was 1819 in the intervention group and 1324 in the control group. The mean number of records per participant was higher in the intervention group than in the control group. Music therapy was the most frequently used intervention in the intervention group, demonstrating the highest usage time (mean 38.21, SD 33.46 min) and engagement level (mean 3.84, SD 0.91). Although exercise therapy had a shorter average usage time (23.12, SD 15.37 min), it recorded the second-highest frequency of use (94/357, 26.3%). Reminiscence therapy had a relatively low usage frequency (82/357, 23.0%) and engagement (mean 3.49, SD 1.03). However, its average usage time exceeded the overall intervention mean of 31.40 (SD 28.79) min. In contrast, reminiscence therapy with music showed the lowest usage frequency (29/357, 8.1%) and shortest average usage time (12.76, SD 9.14 min); however, it received a relatively positive engagement rating (mean 3.69, SD 0.85).

**Table 5.** Summary of behavioral and psychological symptoms of dementia diary records.

	Control	Intervention
Total number of records	1324	1819
Number of records		
Mean (SD)	77.76 (35.17)	113.81 (166.42)
Range	51‐164	42‐735

**Table 6.** Summary of mobile app-based nonpharmacological intervention usage.

Type of intervention^a^	Frequency, n (%)	Usage time, mean (SD)	Engagement level, mean (SD)
Total	357 (100)	31.40 (28.79)	3.71 (0.93)
Music therapy	152 (42.6)	38.21 (33.46)	3.84 (0.91)
Exercise	94 (26.3)	23.12 (15.37)	3.71 (0.84)
Reminiscence therapy	82 (23.0)	34.88 (30.90)	3.49 (1.03)
Reminiscence with music	29 (8.1)	12.76 (9.14)	3.69 (0.85)

a Intervention usage data were collected only for the intervention group.

Exploratory analyses were conducted to examine the relationship between intervention dose and changes in outcome variables. Participants were categorized into high-dose and low-dose groups based on the median values of usage metrics (total frequency and mean usage time). Dose-response exploratory plots are presented in Multimedia Appendix 1. Exploratory comparisons showed no significant differences in pre-post changes of outcome variables between participants with higher and lower intervention usage. This result remained consistent even among participants with baseline NPI scores ≥11.

## Discussion

### Principal Results

Unlike previous studies that primarily implemented clinician-led interventions or were conducted in institutional settings, this study assessed a fully caregiver-driven app-based intervention in real-world community environments. Family caregivers were able to independently monitor symptoms and deliver tailored nonpharmacological interventions at home. In this way, this study demonstrated the feasibility of a decentralized, technology-enabled dementia care model. This approach aligns with the growing demand for accessible and scalable solutions that reduce the reliance on formal care systems. No statistically significant differences were observed between the intervention and control groups in overall BPSD, agitated behavior, or depression after the intervention. However, an exploratory subgroup analysis of participants with more severe BPSD (NPI≥11) suggested a potential benefit (*β*=–12.885; *P*=.03). Given the pilot nature of this study, these findings should be regarded as preliminary and exploratory and not interpreted as definitive. Nevertheless, even mild reductions in BPSD can have clinical significance, as they may help reduce caregiver burden, delay institutionalization, and improve quality of life for both persons living with dementia and their families.

### Comparison With Prior Work

Several factors may account for these small effects. First, regular assessment and documentation of BPSD, including symptom type, severity, and triggers, has been widely recognized as an effective strategy for managing these symptoms [29,50-54]. However, previous studies have often relied on digital databases, such as electronic medical records, which rely on retrospective documentation with limited contextual detail [55,56]. In contrast, the mobile app was used in this study to collect real-time, caregiver-reported data in the home setting. This approach allowed for more precise identification and characterization of BPSD and their triggers as they occurred. The NPI, a commonly used tool for assessing BPSD, has been adapted for daily diaries in mobile apps, minimizing recall bias and enabling users to record and monitor symptoms in real time [52,53]. This tool facilitates easier access to symptom information and enhances symptom awareness, offering therapeutic value through improved recognition and monitoring [53]. In this study, both the intervention and control groups were instructed to assess and document BPSD, including the type, frequency, and severity, using a mobile app modeled after the NPI. Moreover, family caregivers were asked to record potential triggers of BPSD, such as pain, discomfort, or environmental stressors. This approach is consistent with the Need-driven Dementia-compromised Behavior model [57], which views BPSD as an expression of unmet needs triggered by proximal factors. By regularly recording these symptoms and their potential triggers, family caregivers may be better equipped to recognize and preempt these unmet needs. This allows them to address potential issues early, before they escalate into more severe behavioral manifestations [58]. Overall, systematic documentation of BPSD and their triggers via a mobile app serves not only as a real-time monitoring tool but also as a key component of proactive, person-centered dementia care. Such an approach, therefore, aligns with dementia care frameworks, which emphasize that comprehensive assessment should guide tailored nonpharmacological strategies [54].

Second, it is important to consider the individual abilities of persons living with dementia when applying nonpharmacological interventions. Factors such as sensory function, attention span, and mobility limitations can significantly influence the appropriateness and effectiveness of the intervention [59]. To address individual needs, our study included 4 types of mobile app interventions, allowing for flexible selection according to user characteristics. We also made efforts to incorporate individual preferences into the intervention planning process, as tailoring individual preferences may assist in treatment adherence [58]. Preferences were continuously updated based on caregivers’ observations of persons living with dementia’s responses during the sessions. However, it is worth noting that the tailoring efforts in this study relied primarily on caregiver-reported observations of preferences rather than direct input from the persons living with dementia. To complement these efforts, the difficulty level of each activity could be adjusted according to the user’s cognitive and physical function to ensure appropriate challenge and accessibility. In our app, individualized multimedia content such as photos, videos, and music reflecting users’ memories and preferences was incorporated. Future updates could further expand this feature to enhance engagement and emotional connection. This can also be achieved by analyzing and incorporating in-app records, including caregiver-entered engagement data and log data on intervention use. Furthermore, integrating external factors, including real-time data from wearable sensors, environmental monitoring, or smart home technologies, may further improve the adaptability of the intervention [60,61].

Finally, unlike traditional nonpharmacological interventions, which typically maintain a consistent intensity based on the facilitator’s planned structure [15-17], our approach allowed greater flexibility. Participants initiated sessions according to their own preferences. This flexible delivery model may have led to variability in outcomes and how participants engage with the program [62,63]. Consistent with this design feature, we observed considerable variation in usage metrics, including total usage days, number of sessions, and average duration per session. These findings indicate differences in intervention fidelity, which may explain the heterogeneous use patterns observed across different components of the program, including music and reminiscence therapy. Music therapy was the most frequently used intervention in this study, likely due to its ease of use and flexible format [64]. Despite its popularity, however, it did not lead to significant reductions in BPSD. This finding echoes prior findings that family-delivered music interventions show limited effects, whereas those provided by trained professionals yield clearer benefits [33]. In contrast, reminiscence therapy in our study showed the lowest levels of usage and engagement, possibly reflecting greater usability challenges than those associated with music- or exercise-based interventions. Yet, recent evidence indicates that digital reminiscence therapy can be more effective than traditional formats [65].

Our exploratory analyses showed no significant differences between higher- and lower-usage participants, indicating that dosage alone may not drive clinical effects. A previous study reported that factors such as intervention quality, caregiver capacity, and contextual support likely play a more critical role in shaping outcomes [66]. In institutional settings, professional caregivers such as nurses can integrate cognitive stimulation activities into daily routines. By contrast, in home environments, limited expertise and resources among family caregivers may reduce intervention fidelity and impact [67]. To address this, structured support should be provided in community settings, so that caregivers can use the app independently while still receiving regular guidance from trained professionals or volunteers [58,68]. Such support, together with age-friendly interface improvements and user education, may help enhance digital competencies and promote more consistent engagement. These improvements, in turn, could increase the effectiveness of caregiver-initiated, app-based nonpharmacological interventions [66,68]. Broader contextual factors, such as differences in broadband access, digital literacy, and cultural familiarity, may also affect how interventions are used and how well they work. In particular, the fact that many caregivers in our study were older adults may have influenced their ability to consistently use the app. Therefore, tailored implementation strategies are needed for different care settings [66].

### Implications for Practice and Policy

This study provides preliminary evidence on the potential of caregiver-driven, technology-enabled dementia care within the Korean context. The findings suggest that integrating structured guidance and support could enhance caregiver capacity, ensuring a more effective and sustainable implementation of nonpharmacological interventions in home settings. Given the global rise in the prevalence of dementia and reliance on informal caregivers, these exploratory results may inform future efforts to adapt and refine digital intervention frameworks.

### Future Research

Building on the current findings, future studies should consider larger and more diverse samples and examine the effects of stratifying participants according to baseline BPSD severity. Moreover, the development of adaptive personalization algorithms that incorporate direct and continuous monitoring of persons living with dementia’s responses may enhance intervention responsiveness and reduce caregiver burden. Such systems should also dynamically adapt to factors such as modality of use, thereby revealing intervention preferences and informing recommendations for optimal dosage. In addition, tailoring recommendations based on cognitive and physical function as well as BPSD severity may further refine the personalization of nonpharmacological interventions. Finally, evaluating the implementation of this intervention in long-term care or rural settings may extend its utility and scalability. The BPSD diary itself may also serve as a monitoring strategy that helps alleviate symptoms. Therefore, a 3-arm randomized controlled trial including (1) no diary and no intervention, (2) diary monitoring only, and (3) diary monitoring with the intervention could provide robust evidence on their individual and combined effects.

### Limitations

Despite its strengths, this study had several limitations. First, the sample size was relatively small and restricted to urban community-dwelling participants in South Korea, which may limit the generalizability of the findings. While South Korea is one of the most digitally advanced countries [69], the study participants were predominantly from urban areas. As such, the observed feasibility and engagement levels may not fully reflect outcomes in populations with limited broadband access or in rural settings where digital resources are less developed. In addition, the sample size was calculated a priori based on a medium effect size. However, the wide confidence intervals raise the possibility of a type II error and suggest that the study may have been underpowered to detect smaller-than-expected effects. Accordingly, these findings should be regarded as preliminary and interpreted with caution. Second, the personalization of interventions was based on caregiver-reported observations, with limited direct input from persons living with dementia. This reliance on proxy reports may have introduced reporting bias and restricted the extent to which the intervention reflected the lived experiences and preferences of persons living with dementia themselves. Finally, nighttime sleep efficiency, included as a secondary outcome, was already high at baseline (94%), which is comparable to or even higher than levels reported in previous studies involving persons living with dementia [44]. This suggests that participants in our study exhibited relatively preserved sleep quality at baseline. A potential ceiling effect may therefore have constrained the magnitude of observable change. As a result, the interpretation of sleep-related outcomes should take this limitation into account.

### Conclusions

This study evaluated a mobile app–based individualized nonpharmacological intervention for managing BPSD in community-dwelling persons living with dementia. Although improvements were observed, they did not reach statistical significance in the overall analysis. Exploratory findings indicated potential benefits for participants with more severe baseline BPSD; however, these results should be interpreted with caution. Nevertheless, the findings highlight the potential value of empowering family caregivers to deliver flexible, person-centered care at home through digital tools. Future research should refine personalized strategies and optimize intervention delivery by using usage data. Despite its limitations, including a small sample size and variability in engagement, this study shows the potential of caregiver-driven, app-based, individualized nonpharmacological intervention for BPSD in the home and community setting. Continued efforts to integrate digital interventions within community-based care frameworks, accompanied by education and guidance for family caregivers, may contribute to the development of sustainable person-centered dementia care approaches.

## Supplementary material

10.2196/79469 Multimedia Appendix 1 Baseline-post changes according to total frequency and mean usage time within the intervention group.

10.2196/79469 Checklist 1 CONSORT-eHEALTH checklist (V 1.6.1).

## Abbreviations

BPSD: behavioral and psychological symptoms of dementia
NPI: Neuropsychiatric Inventory
