# Supplementary material for: A Mobile App–Based Individualized Nonpharmacological Intervention for Behavioral and Psychological Symptoms in Dementia: Pilot Randomized Controlled Trial
Source: JMIR Mhealth Uhealth. 2026 Apr 7;14:e79469. doi: 10.2196/79469 (PMC13055958; doi:10.2196/79469)
Supplement: Multimedia Appendix 1 [file mhealth-v14-e79469-s001.docx]

**Supplementary 1. Baseline–post changes according to total frequency within the intervention group**

| **Total intervention group (N=16)** | **Intervention group with NPI ≥ 11 at baseline (N=13)** |
| --- | --- |
| 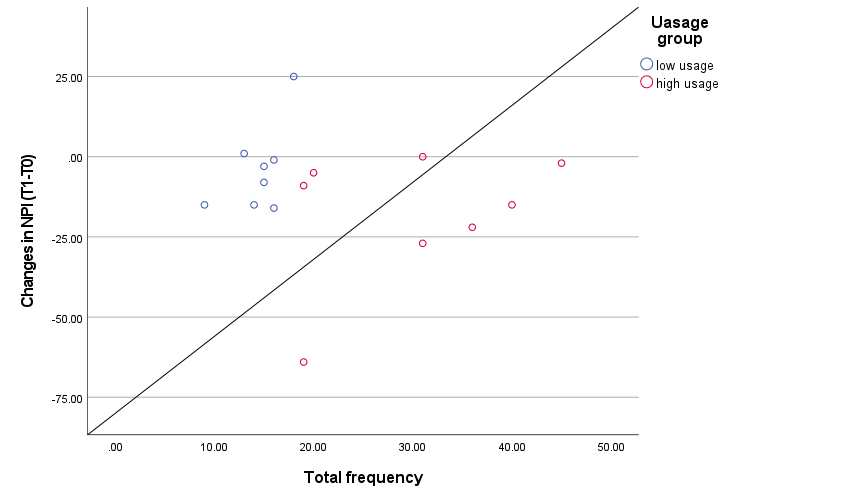 | 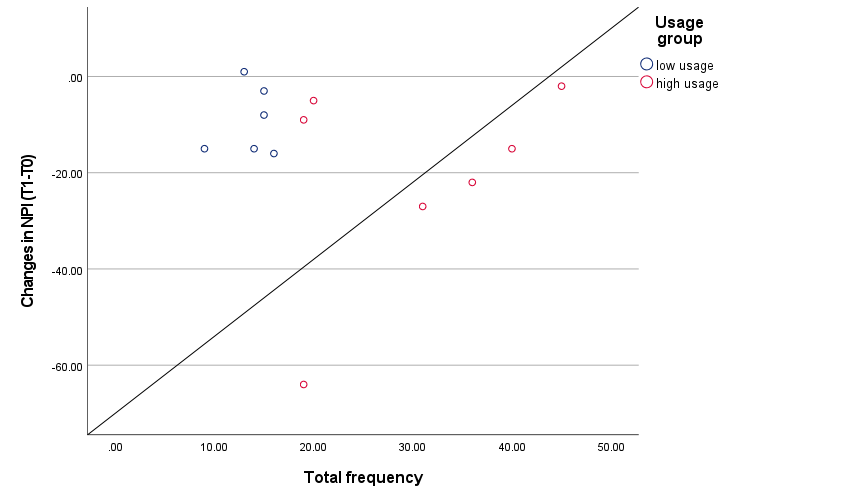 |
| 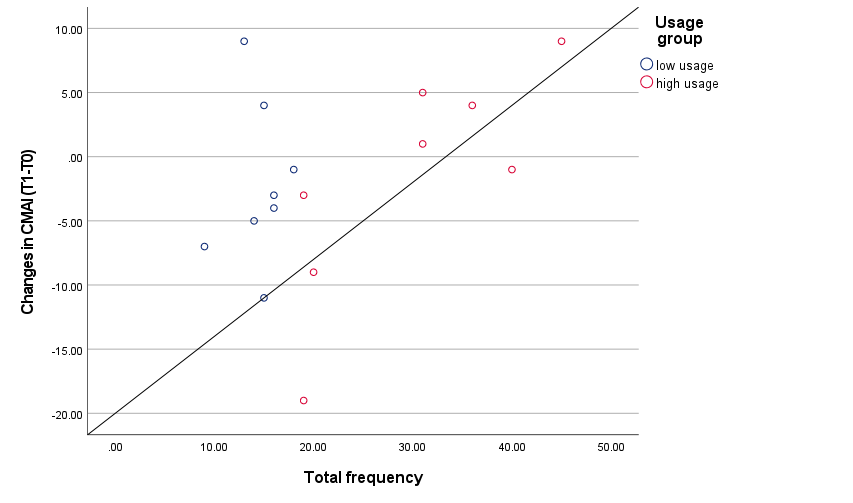 | 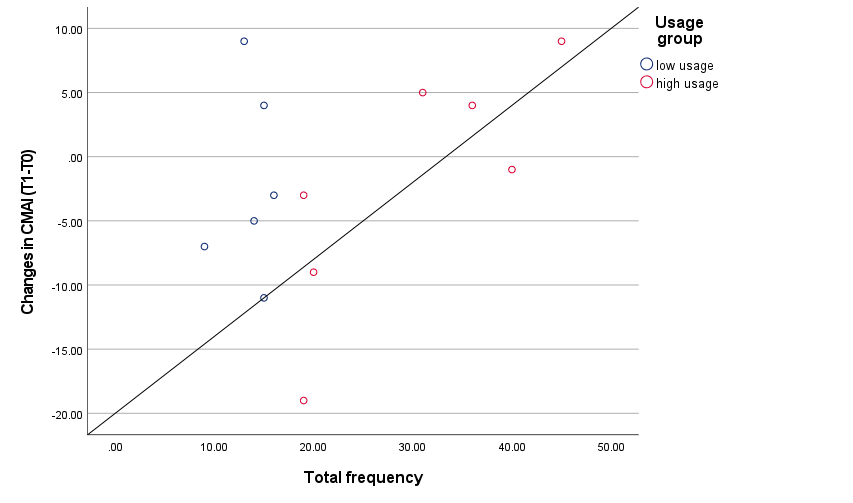 |
| 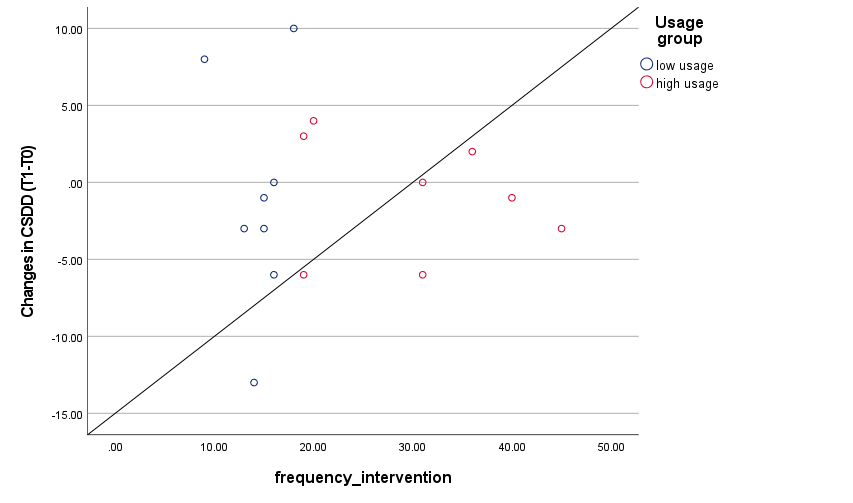 | 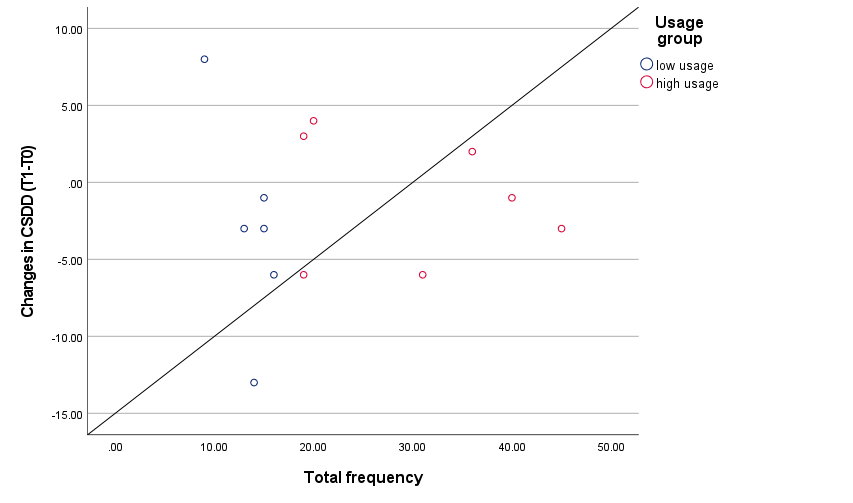 |
| \| **Outcome**  **variables** \| **Low usage (n=8)** \| **High usage (n=8)** \| ***Z(P)*** \| \| --- \| --- \| --- \| --- \| \| **Overall BPSD** \| \| \| \| \| T0^a^ \| 26.63(20.83) \| 34.75(21.85) \| -0.893(.38) \| \| T1^b^ \| 22.63(20.93) \| 16.75(13.37) \| -0.263(.80) \| \| Change \| -4.00(13.51) \| -18.00(20.88) \| -1.264(.23) \| \| Z(*P*) \| -1.193(.23) \| -2.366(**.02**) \|  \| \| **Agitated behavior** \| \| \| \| \| T0 \| 44.50(16.34) \| 47.88(17.68) \| -0.158(.88) \| \| T1 \| 42.25(20.16) \| 46.25(18.33) \| -0.791(.44) \| \| Change \| -2.25(6.30) \| -1.63(8.90) \| -0.632(.57) \| \| Z(*P*) \| -1.051(.29) \| -0.140(.88) \|  \| \| **Depression** \| \| \| \| \| T0 \| 10.00(6.07) \| 8.50(3.02) \| -0.053(.96) \| \| T1 \| 9.00(5.60) \| 7.63(4.60) \| -0.212(.88) \| \| Change \| -1.00(7.37) \| -0.88(3.87) \| -0.212(.88) \| \| Z(*P*) \| -0.508(.61) \| -0.594(.55) \|  \| | \| **Outcome**  **variables** \| **Low usage (n=6)** \| **High usage (n=7)** \| ***Z(P)*** \| \| --- \| --- \| --- \| --- \| \| **Overall BPSD** \| \| \| \| \| T0 \| 33.17(19.96) \| 39.29(19.10) \| -0.787(.45) \| \| T1 \| 23.83(22.45) \| 18.71(13.14) \| 0.000(.99) \| \| Change \| -9.33(7.17) \| -20.57(21.14) \| -1.006(.37) \| \| Z(*P*) \| -1.997(.05) \| -2.366(.02) \|  \| \| **Agitated behavior** \| \| \| \| \| T0 \| 47.83(17.87) \| 50.43(17.43) \| -0.143(.95) \| \| T1 \| 45.67(22.65) \| 48.43(18.65) \| -0.647(.53) \| \| Change \| -2.17(7.39) \| -2.00(9.54) \| -0.359(.73) \| \| Z(*P*) \| -0.734(.46) \| -0.254(.80) \|  \| \| **Depression** \| \| \| \| \| T0 \| 12.00(5.69) \| 9.14(2.61) \| -0.649(.53) \| \| T1 \| 9.00(5.83) \| 8.14(4.71) \| 0.000(.99) \| \| Change \| -3.00(6.84) \| -1.00(4.16) \| -0.651(.53) \| \| Z(*P*) \| -1.156(.25) \| -0.594(.55) \|  \| |

Note: Values are presented as mean (SD)

^a^T0: baseline; ^b^T1: after 4 weeks

**Supplementary 2. Baseline–post changes according to mean usage time within the intervention group**

| **Total intervention group (N=16)** | **Intervention group with NPI ≥ 11 at baseline (N=13)** |
| --- | --- |
| 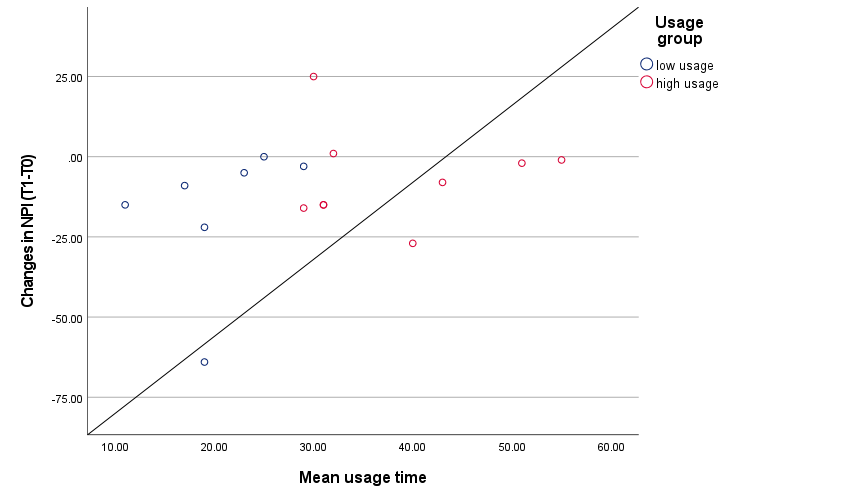 | 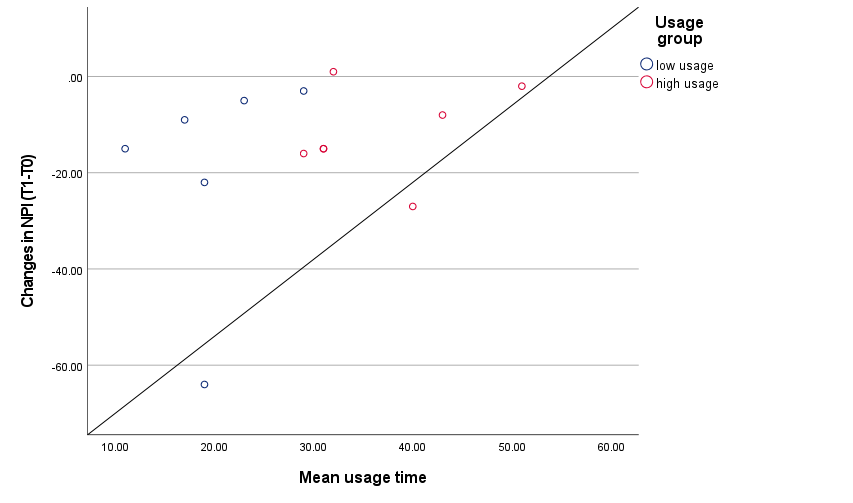 |
| 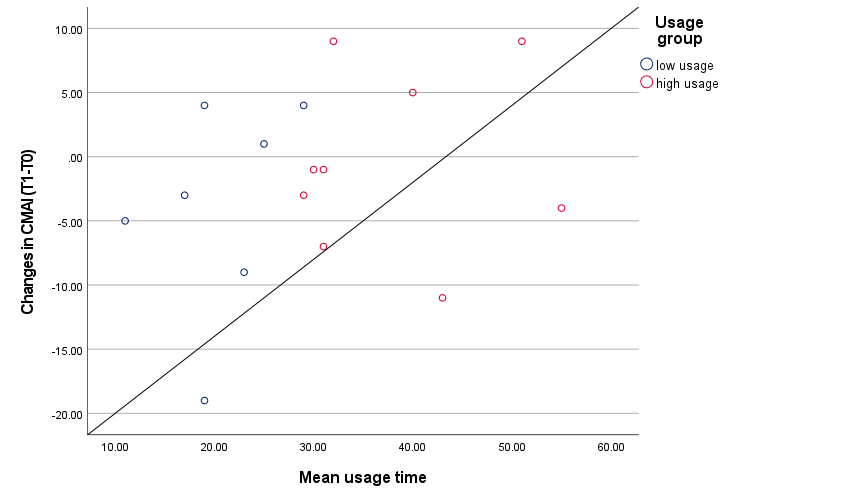 | 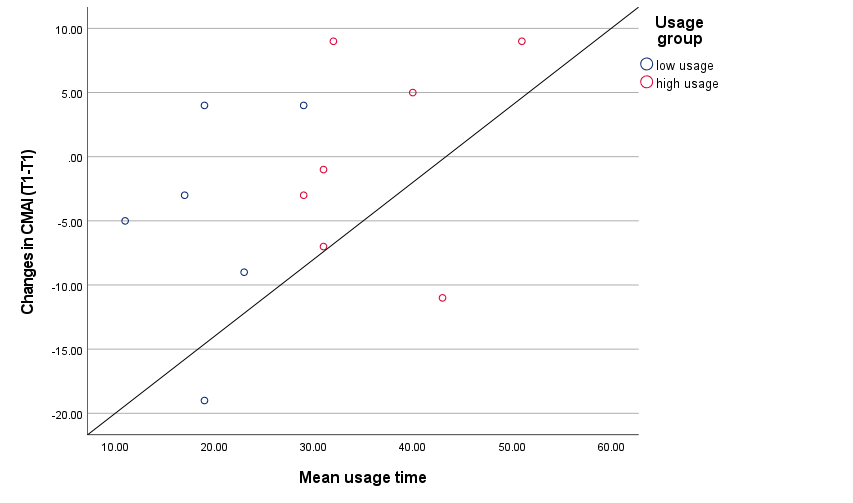 |
| 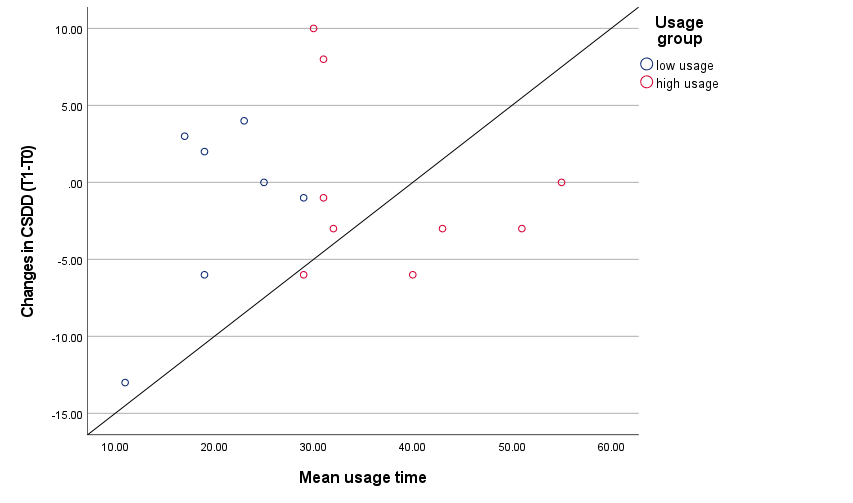 | 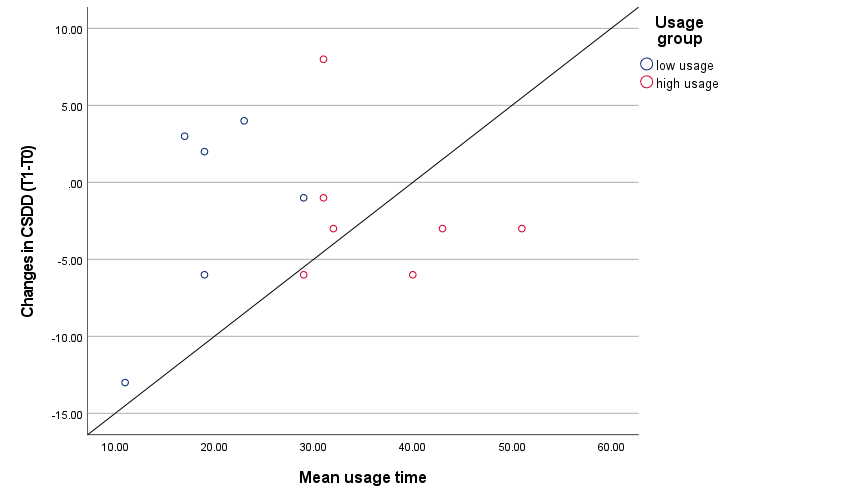 |
| \| **Outcome**  **variables** \| **Low usage (n=7)** \| **High usage (n=9)** \| ***Z(P)*** \| \| --- \| --- \| --- \| --- \| \| **Overall BPSD** \| \| \| \| \| T0 \| 34.86(22.73) \| 27.44(20.39) \| -0.742(.47) \| \| T1 \| 18.00(14.14) \| 21.00(20.06) \| -0.053(.99) \| \| Change \| -16.86(22.10) \| -6.44(14.82) \| -0.690(.54) \| \| Z(*P*) \| -2.201(.03) \| -1.543(.12) \|  \| \| **Agitated behavior** \| \| \| \| \| T0 \| 46.14(15.40) \| 46.22(18.30) \| -0.106(.92) \| \| T1 \| 42.29(14.04) \| 45.78(22.44) \| -0.372(.76) \| \| Change \| -3.86(8.21) \| -0.44(6.91) \| -0.743(.47) \| \| Z(*P*) \| -1.016(.31) \| -0.297(.77) \|  \| \| **Depression** \| \| \| \| \| T0 \| 10.00(4.55) \| 8.67(5.00) \| -0.854(.41) \| \| T1 \| 8.43(5.09) \| 8.22(5.24) \| -0.160(.92) \| \| Change \| -1.57(6.02) \| -0.44(5.73) \| -0.267(.84) \| \| Z(*P*) \| -0.314(.75) \| -0.423(.67) \|  \| | \| **Outcome**  **variables** \| **Low usage (n=6)** \| **High usage (n=7)** \| ***Z(P)*** \| \| --- \| --- \| --- \| --- \| \| **Overall BPSD** \| \| \| \| \| T0 \| 40.17(19.58) \| 33.29(19.29) \| -0.787(.45) \| \| T1 \| 20.50(13.69) \| 21.57(21.20) \| -0.501(.63) \| \| Change \| -19.67(22.80) \| -11.71(9.52) \| -0.431(.73) \| \| Z(*P*) \| -2.201(.03) \| -2.201(.03) \|  \| \| **Agitated behavior** \| \| \| \| \| T0 \| 48.83(14.96) \| 49.57(19.66) \| -0.143(.95) \| \| T1 \| 44.17(14.39) \| 49.71(24.30) \| -0.144(.95) \| \| Change \| -4.67(8.69) \| 0.14(7.82) \| -1.076(.30) \| \| Z(*P*) \| -1.156(.25) \| -0.000(.99) \|  \| \| **Depression** \| \| \| \| \| T0 \| 11.00(4.05) \| 10.00(4.90) \| -0.793(.45) \| \| T1 \| 9.17(5.15) \| 8.00(5.29) \| -0.288(.84) \| \| Change \| -1.83(6.55) \| -2.00(4.76) \| -0.506(.63) \| \| Z(*P*) \| -0.314(.75) \| -1.194(.23) \|  \| |

Note: Footnote as Supplementary 1
